# Multiplication of Dentists

**Published:** 1844-12

**Authors:** 


*Multiplication of Dentists.*—At the present r ae of increase, the dentists will out-number the physicians. There are nine in one short street in Boston, and how many more there are from Roxbury to Winnisimmit Ferry, the Directory does not mention. They all assert, likewise, that the demand for dentistry is increasing. This must undoubtedly arise from the bad work of some of the craft. It takes one half of them to repair the poor operations performed by the rest. Their income, too, quite outvies the charges of the profession. High as their fees are, the public bear the burden without wincing—which proves that a competent man may get his own price for any undertaking. Dental societies are correcting the empiricism of the profession a little, but not quite fast enough. Persons who never knew the composition of an artificial tooth, open an office, puff themselves, and gather customers as successfully as some of the best-taught graduates of the Baltimore College. If the abuse of dentistry could be corrected, the increase of numbers would create no alarm.

